# His Relations with the Teachers, Parents, and Children

**Published:** 1913-05-24

**Authors:** 


### THE SCHOOL DOCTOR.

### IV.—His Relations with the Teachers, Parents, and Children.

It is highly desirable that the school doctor should work in cordial co-operation with the various and often conflicting interests that he finds in his sphere of work. In the first place, he should bear in mind that medical inspection, though a desirable adjunct to education, is after all merely an adjunct; the school exists mainly for the proper teaching of the children, and the head-teacher is, and must be, the supreme authority. Similarly in a class the class teacher is the responsible authority. The school doctor should uphold that authority and show that he recognises it so far as school routine is concerned. He should not needlessly interfere with the class work, or demand from the teachers more time and work than they may be legitimately asked to give. It is, of course, a great convenience for the doctor in examining the children to have the class-teacher present, but it is not always easy to arrange for the teacher to be in the room while the inspection is proceeding. Whatever information the doctor desires with regard to the intelligence or school habits of individual children can be noted on the cards before the inspection commences, and in particular cases the teacher can be referred to without unduly disturbing his routine work. Most head-masters take an interest in the medical examination and contrive to be present at it, sometimes at some personal inconvenience to themselves. Teachers in the elementary schools are already overburdened with work, and unless the doctor is tactful there is a possibility that co-operation may not go so smoothly as it might. Whenever a child is excluded for some contagious defect or some other condition that temporarily unfits him for school work, the head-teacher should be notified, verbally or in writing, by the doctor; it is his duty, and not that of the doctor, to notify the inspectors and divisional superintendents that the child is absent from school. Such exclusion means a loss of grant under certain conditions, and it gives rise, usually, to a great deal of clerical work which is thrown upon the head-teacher.

Co-operation with the parents is highly necessary if school inspection is to be a safeguard and a useful auxiliary instead of a farce. Local conditions must guide the doctor's policy in each case. Sometimes it is a good plan to give a short talk to the parents about the scope and necessity for such inspection; they do not all understand what medical inspection means, and sometimes are apt to look upon it as a needless interference with the liberty of the parent. When its importance is explained, it will usually be found that the parents can be made to assist materially in getting defects treated with the least possible delay. A good nurse is a most helpful assistant in dealing with parents; but the best of all possible nurses does not warrant the school doctor in neglecting to become personally acquainted with the parents and showing them, whenever it is possible to do so, that he endeavours to work not solely in the interests of a central authority but in those of the children and the community as well.

A point often overlooked in regard to co-operation is the attitude of the children themselves. Experience shows that in many instances delays in treatment are due not to the inability or disinclination of the parents to obtain such treatment, but to the objections to treatment of the children themselves. In the case of dental defects this objection is sometimes very strong, and the school doctor has to take it into consideration as a factor which militates against the efficiency of the work done. It is useless to deal with the parents alone. In such cases the children are generally spoilt; they get their wish in most things at home, and the father or mother gives in to them on every point. In such circumstances the school doctor will obtain excellent results by taking the children into his confidence, simply and clearly telling them about the necessities of their case, and illustrating, if possible, what benefit treatment will give them by referring to children who have been treated. The school doctor, indeed in the ideal sense, ought to be the friend of the children, and if he visits his schools regularly and takes an interest in the children, in their games as well as in their class work, he will speedily find that they lose their nervousness in his presence and readily follow his instructions. Here, again, tact and thoughtfulness are demanded. It is useless to tell a child who is to undergo an operation for adenoids that he will not suffer any pain or inconvenience; the right way is to appeal to his manliness and point out that the pain or inconvenience is temporary and that the benefits of the treatment far outweigh any temporary inconvenience. When this is done it is striking to observe how the percentage of cases treated increases; the children become allies of the doctor instead of strong opponents, and the parents quickly note the fact that the influence of the school doctor is all for good.

Considerable tact is also demanded in the school doctor's relations with his professional colleagues in the district in which he works. The ordinary rules of medical etiquette—which may be summarised into the adage "Do unto others as you would like others to do unto you"—are sufficient to guide the school doctor's conduct in the majority of cases. Thus, the medical inspector should refrain from commenting to the parents on the treatment given in certain cases by hospital or private practitioners. Even where he considers such treatment has been unsound, he should merely note the fact and tell the parent that further treatment is desirable. In many cases it is wise to point out that there are certain difficulties which cannot be overcome by one operation or that a prolonged course of after-treatment is necessary in order to obtain the full benefits of a certain operation. It is especially necessary that the treatment given by the family practitioner should be banned for discussion with the parents; remarks thoughtlessly made by the school doctor, even with the best intentions in the world, are often distorted and misrepresented, with the result that the general practitioner comes to look upon his colleague who does school work as an active enemy who is undermining his reputation and influence in a particularly insidious way. It is undoubtedly true that very often cases arise in which a conflict of opinion is unavoidable. Then the school doctor must use his discretion.

Two cases may serve as illustration. In one a schoolboy was seen and his vision noted as R 24; L 18; the mother was told that he probably wanted glasses and that in any case his eyes needed further examination. At the reinspection, six months later, the defect had not been treated, and the child produced a certificate from his family doctor to the effect that "this child suffers from slight astigmatism, for which no glasses are required."

On inquiry, it was found that no attempt had been made to estimate the refraction either by retinoscopy or by the use of a mydriatic. The child was tested again with the Snellen card and his defect was pointed out to him, it being explained that unless he were given spectacles his vision would probably deteriorate. His mother was told to take him back to his own doctor and ask for further examination. This was done, largely owing to the importunity of the boy himself, and the practitioner advised a call at an eye hospital to take a third opinion, where he was given spectacles. In the other case a child, also a boy, wore an ill-fitting truss for a fairly large inguinal epiplocele; as the child was very active and was a suitable subject for a radical cure, his parents were advised that further treatment was necessary. They reported that their family doctor strongly objected to any operation and had recommended a new truss. This was found to be equally useless, and the advantages of operation were pointed out to the father, who was present at the inspection, it being again urged that he should discuss the case fully with his doctor. As a result a radical operation was performed, with excellent results. In both these cases the difference between the school doctor and the family practitioner was a radical one, and in both stress was laid on the importance of consulting again with the family doctor before deciding as to treatment. Probably the opinion given in the second case was incorrectly reported, and it would be a help to the school doctor if the general practitioner stated his opinion in certain cases, in confidence, for the guidance of his colleague. In the future such co-operation will no doubt be a matter of routine; but at present it is not, and great care is needed to avoid hurting professional susceptibilities.

